# Optimization of dietary methionine requirements under aflatoxicosis in quail chicks using response surface modeling

**DOI:** 10.1016/j.psj.2025.106243

**Published:** 2025-12-10

**Authors:** Mehran Mehri, Morteza Asghari-Moghadam, Mahmoud Ghazaghi, Amir Karamzadeh-Dehaghani, Mohsen Amraie, Mohammad Rokouei

**Affiliations:** a Department of Animal Sciences, Collage of Agriculture, University of Zabol, Zabol 98661-5538, IRAN; b Department of Animal Sciences, Campus of Agriculture and Natural Resources, University of Tehran; Karaj 77781-31587, IRAN; c Department of Animal Sciences, Agriculture Faculty, Lorestan University, Lorestan, IRAN

**Keywords:** Aflatoxin B₁, Immunity, Methionine optimization, Quail, Response surface model

## Abstract

This study aimed to determine the optimal dietary methionine (Met) requirement for quail chicks exposed to different concentrations of aflatoxin B₁ (AFB) using response surface and quadratic polynomial modeling. Previously, excess Met was shown to alleviate the adverse effects of AFB on performance, liver function, and immunity in quail. Here, the same experimental dataset was reanalyzed to quantify Met–AFB interactions and define nutrient optima. Three levels of dietary Met (5.0, 6.0, and 7.0 g/kg) and three levels of AFB (0.0, 2.5, and 5.0 ppm) were modeled across performance, biochemical, immunological, and microbial variables. Model fits were high (R² = 0.62 to 0.95). Increasing dietary Met improved feed intake, gain, feed conversion ratio, and immune indices (hemagglutination inhibition and SRBC titers) while reducing serum hepatic enzymes and malondialdehyde concentration. Methionine supplementation also counteracted AFB-induced dysbiosis by increasing lactic acid bacteria and restoring *E. coli* balance. The optimized models indicated that dietary Met requirements increased proportionally with aflatoxin exposure, reaching approximately 130–150 % of the NRC (1994) recommendation to maintain performance, hepatic integrity, and immune resilience. These results demonstrate that Met acts as both a structural and functional nutrient under toxin stress. The response surface approach provides a quantitative framework for formulating Met -enriched diets for poultry exposed to mycotoxins.

## Introduction

Aflatoxin B₁ (AFB), a potent secondary metabolite produced by *Aspergillus* species in contaminated feed, poses a substantial threat to the productivity and health of poultry worldwide, particularly under hot and humid conditions favoring fungal growth. Quail chicks are among the most susceptible avian species, exhibiting impaired growth, compromised hepatic function, depressed immune responses, and disrupted intestinal epithelial barrier when exposed to AFB (Bagherzadeh-Kasmani et al., 2015; Ghorbani et al., 2025; Khanipour et al., 2019). The detrimental impacts arise primarily through the induction of oxidative stress, hepatocellular injury, and microbiota dysbiosis, as well as reduced nutrient absorption and intestinal integrity.

Methionine (Met) is an essential sulfur-containing amino acid fundamental to protein synthesis, methyl-group transfer, and antioxidant defense mechanisms. Recent studies have highlighted Met's dual role as both a structural and functional nutrient in poultry diets, especially under conditions of oxidative or toxic stress (Ghorbani et al., 2025). Methionine supplementation above basal requirements has been shown to mitigate the adverse consequences of AFB exposure, improving growth performance, restoring hepatic enzyme profiles, boosting immune indices, and ameliorating gut microbial imbalances in various poultry species, including quail (Ghorbani et al., 2025) and broilers (Singh et al., 2013).

Despite evidence for the protective effect of excess dietary Met, the precise requirement for methionine under escalating levels of AFB contamination remains inadequately defined. Predictive, quantitative modeling is essential to address nutrient–toxin interactions and optimize dietary formulations under stress conditions. Response surface methodology (RSM) and quadratic polynomial models have gained recognition as robust tools to explore multidimensional factor effects, estimate nutrient optima, and guide efficient dietary interventions in poultry nutrition (Mehri et al., 2012; Roush et al., 1979).

In our previous study (Ghorbani et al., 2025), we demonstrated that excess dietary Met mitigates the adverse effects of AFB in quail chicks by improving growth, immunity, liver function, and gut microbiota balance. However, while those results established the protective role of Met, the optimal dietary requirement of Met under varying levels of AFB exposure remained undetermined.

Therefore, the present study aimed to quantitatively model and optimize Met levels at different AFB concentrations using quadratic polynomial and response surface analyses. This approach provides a predictive framework for defining Met requirements in birds subjected to mycotoxin challenges.

## Materials and methods

### Dataset

The dataset used in this study originated from our previously published experiment on dietary methionine and AFB interaction in Japanese quail (Ghorbani et al., 2025). The original experiment was conducted in a 3 × 3 factorial arrangement of treatments, consisting of three dietary Met levels (5.0, 6.0, and 7.0 g/kg) and three AFB concentrations (0.0, 2.5, and 5.0 ppm). Details of animal management, experimental design, feed formulation, toxin preparation, and measurement of performance, biochemical, immune, and microbial responses are fully described in Ghorbani et al. (2025). In the present study, those data were reanalyzed using quadratic polynomial and response surface regression models to estimate optimal dietary Met levels (Metₒₚₜ) for each AFB concentration. This reanalysis focused exclusively on statistical optimization and modeling aspects not included in the original publication.

### Statistical analysis

Data were analyzed using the General Linear Model (GLM) procedure in R (version 4.3.3), with cage serving as the experimental unit. Two sets of analyses were conducted. First, as a factorial arrangement of treatments examining the effects of dietary concentrations of Met and AFB and their interaction. The effects were considered significant at *P* ≤ 0.05. Second, if the interaction or main effects were significant, then the parameter estimates for the second-order response surface model were determined using the *lm()* procedure in R. All calculations started with the full quadratic model, but if needed, the model was reduced by removing parameter estimates that were not significant (*P* > 0.05), and the estimates were recalculated using the reduced model, following the method described by González-Vega et al. (2016). Linear and quadratic effects of both Met and AFB and the interaction between Met and AFB were included in the full model as follows:

$$Y=a+b\left(Met\right)+c\left(Met^{2}\right)+d\left(AFB\right)+e\left(AFB^{2}\right)+f\left(Met\;\times \;AFB\right)$$

where Y is the dependent variable, a is the intercept, b, c, d, e, and f are the model coefficients, and Met and AFB are the concentrations of dietary Met (g/kg) and AFB (ppm), respectively.

The concentrations of Met that produced the optimal response values (maximum or minimum depending on the response variable) were calculated using the following equation:

$$Met_{opt}\left(g/kg\right)=\;\frac{\left[-\left(f\;\times \;AFB\right)-b\right]}{2c}$$

where Metₒₚₜ represents the dietary Met concentration (g/kg) associated with the optimal predicted response and AFB is the dietary AFB concentration (ppm). The maximum or minimum predicted response values were then calculated by substituting the Metₒₚₜ values into the respective model equation for each AFB concentration level. In cases where the Met × AFB interaction was not significant, the pooled quadratic regression model was fitted across all AFB levels, and the single optimum Met level (Metₒₚₜ) was calculated from the derivative of the polynomial equation:

$$Met_{opt}\left(g/kg\right)=\;\frac{-b}{2c}$$

where *b* and *c* are the linear and quadratic coefficients of Met, respectively.

## Results and discussion

Quadratic polynomial models were developed to describe the responses of performance, biochemical, immunological, and microbial parameters to dietary Met and AFB levels (Table 1). Model fit was high across all traits, with coefficients of determination (R²) ranging from 0.62 to 0.95. Feed intake, gain, and hepatic enzymes exhibited the strongest model fits (R² ≥ 0.90), whereas microbial variables showed moderate but acceptable predictive power (R² = 0.65–0.74).

**Table 1:** Quadratic polynomial models and coefficients of determination (R²) for broiler responses as affected by methionine (Met) and aflatoxin B₁ (AFB) levels.

Response	Equation	R^2^	Adj_R^2^
F	F = 364.7694 + 14.7533 (Met) - 31.3297 (AFB)	0.91	0.91
G	G = 176.9088 + 2.0906 (Met) - 29.9170 (AFB) + 0.8615 (AFB²)	0.95	0.95
FCR	FCR = 1.9550 + 0.0744 (Met) + 0.3703 (AFB) - 0.0443 (Met × AFB)	0.81	0.79
UA	UA = −48.2501 + 16.3210 (Met) - 1.1917 (Met²) + 1.0770 (AFB) + 0.3003 (AFB²) - 0.4517 (Met × AFB)	0.85	0.82
AST	AST = 511.5279 - 118.9362 (Met) + 9.2508 (Met²) + 40.9662 (AFB) - 5.0375 (Met × AFB)	0.93	0.92
HI	HI = −15.3333 + 7.0833 (Met) - 0.5000 (Met²) - 0.7667 (AFB) + 0.0800 (AFB²)	0.78	0.76
SRBC	SRBC = 29.8750 - 8.2917 (Met) + 0.7917 (Met²) - 0.3167 (AFB)	0.83	0.82
MDA	MDA = 3.9454 - 0.8479 (Met) + 0.0562 (Met²) + 0.0362 (AFB)	0.86	0.85
E.coli	E.coli = 9.0183 + 0.1317 (Met) - 1.5362 (AFB) + 0.0894 (AFB²) + 0.1270 (Met × AFB)	0.74	0.70
LAB	LAB = −9.6796 + 5.5392 (Met) - 0.3900 (Met²) - 0.1582 (AFB)	0.65	0.62

F: feed intake; G: gain; FCR: feed conversion ratio; UA: uric acid; AST: aspartate aminotransferase; HI: hemagglutination inhibition; SRBC: sheep red blood cell; MDA: malondialdehyde; LAB: lactic acid bacteria.

Feed intake and gain increased linearly (*P* < 0.01) with increasing Met, while AFB depressed these responses in a dose-dependent manner. The fitted equations indicated that every 1 g/kg increase in dietary Met raised feed intake by approximately 14.8 g and gain by 2.1 g, while each 1 ppm increase in AFB reduced these variables by 31.3 g and 29.9 g, respectively. Feed conversion ratio (FCR) improved with Met supplementation in AFB-fed birds, consistent with the negative Met × AFB term in the model equation.

Methionine supplementation exerted a protective effect against hepatic damage caused by AFB exposure. The model for aspartate aminotransferase (AST) demonstrated a strong negative coefficient for Met (–118.9) and a positive quadratic term (+9.25), indicating a curvilinear decline in enzyme activity up to the optimum Met level. Similar patterns were observed for uric acid (UA), where a significant quadratic response (Met² = –1.19) reflected a decline in circulating UA concentrations at higher Met levels, particularly under AFB exposure.

Both hemagglutination inhibition (HI) and sheep red blood cell (SRBC) antibody titers increased (*P* < 0.001) with rising dietary Met, while AFB suppressed humoral immunity. The models indicated quadratic responses to Met, with estimated plateaus at approximately 6.0 g/kg Met for maximum antibody production. AFB exerted a negative main effect in both immunity models, but Met supplementation effectively compensated for this suppression, maintaining near-normal antibody titers even at 5.0 ppm AFB.

Methionine supplementation reduced lipid peroxidation, as reflected by declining malondialdehyde (MDA) concentrations with increasing Met (R² = 0.86). The quadratic term (Met² = +0.056) indicated a curvilinear pattern, suggesting maximal antioxidant protection near 6.5 g/kg Met.

AFB decreased lactic acid bacteria (LAB) counts (*P* = 0.015) and disrupted microbial balance, whereas increasing dietary Met improved both LAB and *E. coli* populations toward physiological levels. For *E. coli* populations, the model (R² = 0.74) indicated that AFB had a strong linear inhibitory effect (–1.54), reducing *E. coli* counts as toxin concentration increased. However, the positive interaction between Met and AFB (+0.127) suggested that Met supplementation mitigated this suppression, restoring microbial balance at higher toxin levels. The positive quadratic term for AFB₁ (+0.0894) reflected a slight recovery of *E. coli* at the highest AFB dose, consistent with adaptive microbial stabilization. For LAB, the negative coefficient for AFB (–0.1582) demonstrated a uniform reduction in LAB counts with toxin exposure, regardless of Met level.

**Fig. 1:**
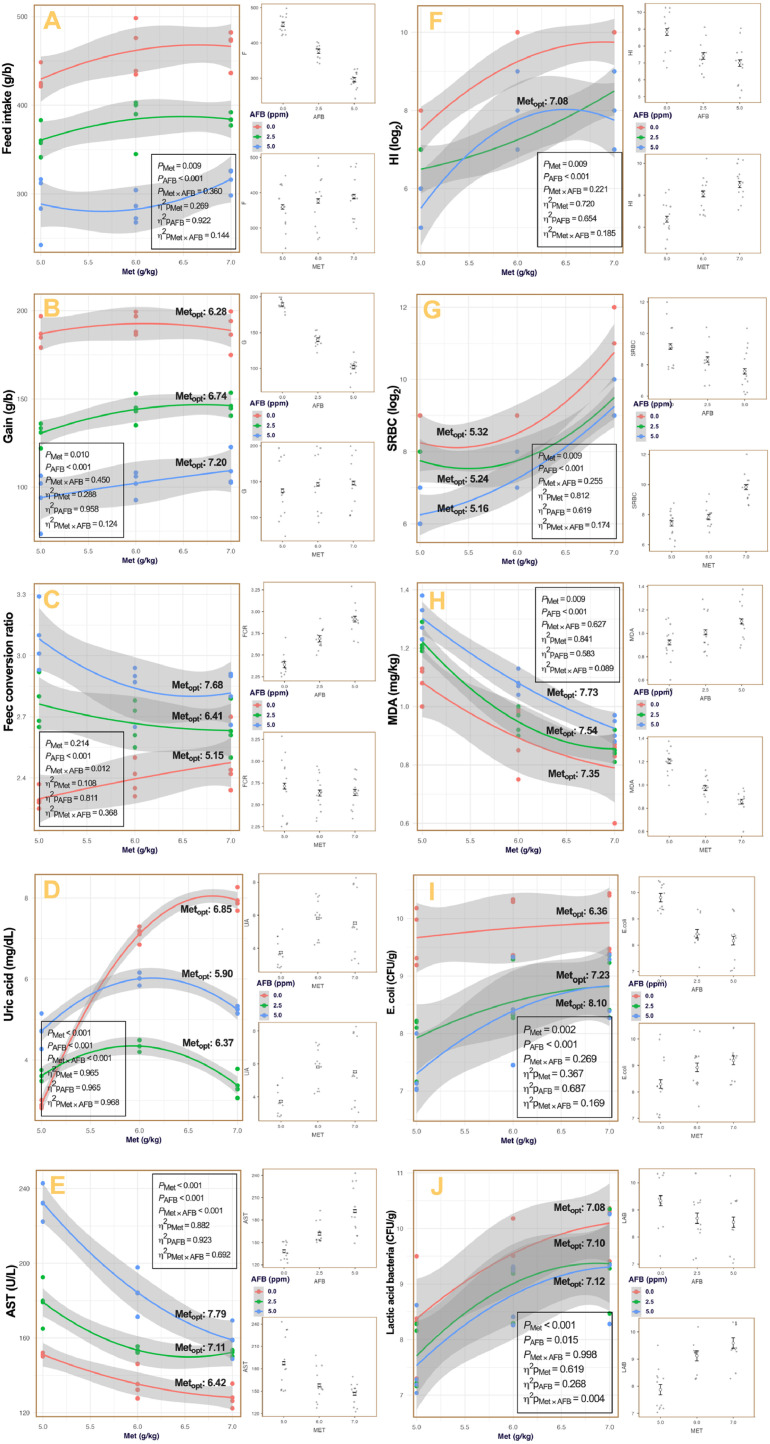
Response of performance (A–C), biochemical markers (D–E), humoral immunity (F–G), oxidative status (H), and intestinal microbiota (I–J) of growing quails to dietary methionine (Met; g/kg) and aflatoxin B₁ (AFB; ppm). Each panel presents fitted quadratic regression curves (±95 % confidence intervals) for three AFB levels (0.0, 2.5, and 5.0 ppm), with corresponding data points overlaid. The estimated methionine concentrations producing optimal predicted responses (Metₒₚₜ) are labeled on each curve. Reported P-values and partial η² represent the significance and effect size of Met, AFB, and Met × AFB interaction terms in the response surface models. Panels represent: A) feed intake, B) gain, C) feed conversion ratio, D) serum uric acid, E) serum aspartate aminotransferase (AST), F) hemagglutination inhibition (HI) titer, G) SRBC antibody titer, H) muscle malondialdehyde (MDA), I) *E. coli*, and J) lactic acid bacteria.

The estimated optimal Met concentrations (Metₒₚₜ) derived from both models and response surface plots are presented in Table 2 and Fig. 1. For growth performance traits, Metₒₚₜ increased with AFB exposure, ranging from 6.3 to 7.2 g/kg for feed intake and 6.3 to 7.2 g/kg for gain, while FCR showed optima between 5.2 and 7.7 g/kg, equivalent to 126–154 % of NRC (1994). Uric acid (UA) displayed an inverse trend, with Metₒₚₜ declining from 6.8 to 5.9 g/kg (137–118 % of NRC) as AFB increased. Hepatic responses followed expected patterns, with AST reaching its minimum between 6.4 and 7.8 g/kg (129–156 % of NRC). For immunological traits, hemagglutination inhibition (HI) was maximized consistently around 7.1 g/kg (≈142 % of NRC), whereas SRBC titers peaked at approximately 5.2 g/kg (≈105 % of NRC). Oxidative status, measured via MDA, showed optimal reduction at 7.3–7.7 g/kg (147–155 % of NRC). Among intestinal microbial populations, Met supported both *E. coli* and lactic acid bacteria (LAB) under AFB challenge, with Metₒₚₜ values ranging from 6.4 to 8.1 g/kg for *E. coli* and stabilizing near 7.1 g/kg for LAB (≈142 % of NRC). Overall, Metₒₚₜ values rose progressively with AFB level, averaging 130–150 % of NRC (1994).

**Table 2:** Optimized methionine requirements of quail chicks exposed to varying doses of aflatoxin B_1_ (AFB) compared to NRC (1994) recommendation.

Response	AFB (ppm)	Objective	Met_opt_ (% of NRC, 1994)
Gain	0.0	Maximize	126
	2.5	Maximize	135
	5.0	Maximize	144
HI	0.0	Maximize	142
	2.5	Maximize	142
	5.0	Maximize	142
SRBC	0.0	Maximize	106
	2.5	Maximize	105
	5.0	Maximize	103
LAB	0.0	Maximize	142
	2.5	Maximize	142
	5.0	Maximize	142
AST	0.0	Minimize	129
	2.5	Minimize	142
	5.0	Minimize	156
FCR	0.0	Minimize	103
	2.5	Minimize	128
	5.0	Minimize	154
UA	0.0	Minimize	137
	2.5	Minimize	127
	5.0	Minimize	118
MDA	0.0	Minimize	147
	2.5	Minimize	151
	5.0	Minimize	155

G: gain; HI: hemagglutination inhibition; SRBC: sheep red blood cell; LAB: lactic acid bacteria; AST: aspartate aminotransferase; FCR: feed conversion ratio; UA: uric acid; MDA: malondialdehyde.

The present study demonstrates that Met supplementation at levels substantially exceeding the NRC (1994) recommendations exerts pronounced protective effects against aflatoxicosis in growing poultry, encompassing performance, biochemical, immunological, and gut health outcomes. The quadratic model, based on growth, hepatic markers, and microbial dynamics, showed strong coefficients (0.62-0.95), supporting the biological relevance of the dosing estimates. The models predicted optimal Met requirements that were significantly higher than NRC (1994) recommended levels, especially under toxin-challenge conditions, reinforcing the necessity to revisit classical benchmarks when formulating diets for birds exposed to nutritional stresses such as mycotoxins.

The NRC (1994) guidelines, derived primarily from studies in toxin-free conditions, recommend dietary Met concentrations of 0.45 – 0.50 % (4.5 – 5.0 g/kg) for optimal growth in broilers and quail. However, mounting evidence indicates that such levels may be insufficient when birds are exposed to oxidative or metabolic stressors. The results of this study underscore a paradigm shift in optimal feed intake, weight gain, and FCR occurred at Met levels significantly above baseline under AFB challenge. For each 1 g/kg increase in Met, intake and gain improved by 14.8 g and 2.1 g, respectively, while every 1 ppm increase in AFB reduced these metrics by over 30 g, effects that were substantially offset by supranutritional Met dosing. These findings corroborate recent work in quail and broilers, where dietary Met at 6.0 –7.0 g/kg not only ameliorated performance loss but restored hepatic enzyme profiles and immune indices closer to baseline (Sharma et al., 2015).

Methionine’s protective effects in aflatoxin-rich environments are mechanistically rooted in its role as a precursor for glutathione (GSH) synthesis and its participation in transmethylation and trans-sulfuration cycles. Increased dietary Met boosted liver GSH and GST in both quail and broiler models. This correlated with lower AST and ALT levels, markers of liver injury. The reduction in serum uric acid and MDA provides further evidence of improved redox homeostasis (Elwan et al., 2021; Sharma et al., 2015). A recent study in Japanese quail demonstrated that Met levels of 6.0 – 7.0 g/kg nearly restored growth and feed efficiency in birds exposed to 2.5 – 5.0 ppm AFB, and attenuated the elevation of hepatic enzyme activities (e.g., AST) significantly compared to standard supplementation (Ghorbani et al., 2025).

Unlike growth and hepatic enzyme responses, which indicated an increased Met requirement with higher AFB exposure, the UA response showed the reverse pattern. As toxin concentration increased, the model predicted a lower Met_opt_ to minimize UA. Main effect plots further confirmed that UA concentration sharply declined at moderate AFB levels and remained lower than in toxin-free birds despite changes in Met supplementation. In healthy birds, higher Met intake promotes amino acid catabolism and uricogenesis, leading to greater nitrogen excretion as UA. As liver function deteriorates, protein degradation and UA synthesis decrease. Concurrently, Met assumes a protective, rather than catabolic, function by providing methyl groups and cysteine for GSH synthesis, which mitigates oxidative damage and supports hepatocyte repair (Elwan et al., 2021). Improved liver detoxification leads to increased nitrogen retention in tissue protein rather than loss as UA. Therefore, under higher AFB exposure, less additional Met is needed to suppress UA because the toxin itself limits uricogenesis and redirects nitrogen toward anabolic recovery. Although the calculated Met level for minimizing serum UA (∼5.9 g/kg) is lower than that required for maximizing weight gain and immune responses (6.3–7.2 g/kg), this difference should be viewed in the context of production goals. The lower Met requirement for UA minimization likely reflects impaired uricogenesis capacity due to liver damage rather than optimal health. Therefore, higher Met levels (around 7.0 g/kg) are recommended, as they maximize economically important traits (growth) and survival indicators (immunity), even if they exceed the theoretical optimum for UA minimization.

Beyond direct antioxidant and detoxification effects, supranutritional Met dosing has shown notable benefits for immune competence, especially under aflatoxin stress. The present dataset and several cited studies found that humoral immune responses, antibody titers against SRBC antigen and hemagglutination inhibition, were not only protected but enhanced at Met levels above NRC (1994) standards. This finding aligns with the amino acid’s role as a methyl-group donor, influencing DNA and histone methylation events that drive immune cell proliferation and cytokine production. Aflatoxins are well documented for their immunosuppressive properties, including lowered lymphocyte counts, reduced cytokine expression, and diminished antibody response, thereby increasing disease susceptibility in poultry. By increasing dietary Met, birds exhibited greater antibody production and restored basal levels of lactic acid bacteria in the ileum, a sign of improved gut immunity and microbial stability.

The regression-based approach used in this study provided a robust and biologically meaningful estimation of optimal dietary Met concentrations at different levels of AFB. Quadratic and response surface models effectively captured both the linear and curvilinear components of Met and AFB interactions, explaining a large proportion of the variation (R² = 0.62–0.95) across traits. The use of polynomial regression allowed the determination of the turning points (e.g., Metₒₚₜ) where further supplementation produced diminishing or adverse returns, thereby identifying the biological plateau in nutrient utilization. In the current dataset, separate quadratic regressions were fitted for each AFB level, and the intersection of the first derivative (dY/dMet=0) provided the point of maximum or minimum response, depending on the variable’s objective (growth maximization or metabolite minimization). When the Met × AFB interaction was not significant, the single optimal Met was estimated from the pooled regression across toxin levels. This statistical approach integrates both nutritional and toxicological factors, allowing simultaneous visualization of performance responses and biochemical perturbations across a multidimensional surface. Such regression models are particularly advantageous for mycotoxin–nutrient interaction studies because they accommodate nonlinear biological responses and the antagonistic effects of toxins on nutrient utilization. Unlike linear dose–response models, polynomial regressions enable prediction of both deficiency and excess effects and are more reflective of the physiological reality where Met acts as both a limiting amino acid and a detoxification cofactor. Consequently, the derived equations provided accurate estimations of the Met requirement under each AFB condition, showing progressive shifts from approximately 5.5 – 6.0 g/kg in toxin-free birds to 6.5 – 7.0 g/kg under 2.5–5.0 ppm AFB. These regression-derived optima align closely with prior response surface studies in broilers and quail that evaluated sulfur amino acid adequacy under oxidative or immune stress (Sharma et al., 2015).

In summary, this study and supporting literature show that NRC (1994) methionine recommendations are inadequate under aflatoxin challenge. Methionine becomes essential not only for growth but also for antioxidant defense, detoxification, immunity, and gut integrity during toxin exposure. Therefore, Met requirements should be recalibrated using context-specific models to maintain performance and health in mycotoxin-prone environments. Supranutritional Met supplementation represents an effective strategy to protect poultry against aflatoxicosis.

## CRediT authorship contribution statement

**Mehran Mehri:** Writing – review & editing, Writing – original draft, Formal analysis, Conceptualization. **Morteza Asghari-Moghadam:** Writing – review & editing, Resources, Project administration. **Mahmoud Ghazaghi:** Writing – review & editing, Investigation, Data curation. **Amir Karamzadeh-Dehaghani:** Writing – review & editing, Software, Funding acquisition. **Mohsen Amraie:** Software, Validation, Writing – review & editing. **Mohammad Rokouei:** Writing – review & editing, Visualization, Validation, Methodology.

## Disclosures

The authors declare that they have no known competing financial interests or personal relationships that could have appeared to influence the work reported in this paper.
